# An Examination of the Multi-Faceted Motivation System in Healthy Young Adults

**DOI:** 10.3389/fpsyt.2018.00191

**Published:** 2018-05-11

**Authors:** Susana Da Silva, Areti Apatsidou, Sarah Saperia, Ishraq Siddiqui, Eliyas Jeffay, Aristotle N. Voineskos, Zafiris J. Daskalakis, Gary Remington, Konstantine K. Zakzanis, George Foussias

**Affiliations:** ^1^Centre for Addiction and Mental Health, Toronto, ON, Canada; ^2^Institute of Medical Science, University of Toronto, Toronto, ON, Canada; ^3^Department of Psychology, University of Toronto Scarborough, Scarborough, ON, Canada; ^4^Department of Psychiatry, University of Toronto, Toronto, ON, Canada

**Keywords:** amotivation, reward system, schizotypal traits, depressive symptoms, RDoC

## Abstract

**Background:** Amotivation is a prevalent symptom in schizophrenia (SZ) and depression (MDD), and is linked to poor functional outcomes in affected individuals. Conceptualizations of motivation have outlined a multi-faceted construct comprised of reward responsiveness, reward expectancy, reward valuation, effort valuation, and action selection/preference-based decision making. To date, findings from studies utilizing variable-centered approaches to examining isolated facets of motivation in SZ and MDD have been inconsistent. Thus, the present study adopted a person-centered approach, and comprehensively examined the reward system in a non-clinical sample in an attempt to explore potential subtypes of motivation impairments, while minimizing the effects of illness-related confounds.

**Methods:** Ninety-six healthy undergraduate students were evaluated for amotivation, schizotypal traits, depressive symptoms, and cognition, and administered objective computerized tasks to measure the different facets of motivation. Cluster analysis was performed to explore subgroups of individuals based on similar motivation task performance. Additionally, correlational analyses were conducted in order to examine inter-relationships between motivation facets, and relations between clinical measures and facets of motivation.

**Results:** Cluster analysis identified two subgroups of individuals with differential motivation performance profiles. Correlational analyses revealed that reward responsiveness was associated with amotivation, depressive symptoms, and negative schizotypy. Further, significant inter-correlations were found between reward responsiveness and reward expectancy, as well as between reward valuation and effort valuation.

**Conclusions:** Our results mark important steps forward in understanding motivation in a non-clinical sample, and guide future dimensional and comprehensive analyses of the multi-faceted reward system. It remains to be seen whether these patterns of results will be similar in clinical populations such as SZ and MDD.

## Introduction

Amotivation, a reduction in the ability to initiate and/or sustain goal-directed behavior, is a prevalent symptom in a number of neuropsychiatric illnesses such as schizophrenia (SZ) and major depressive disorder (MDD), and is inextricably linked to poor functional outcomes in affected individuals (1–4). Recognizing the importance of better understanding this symptom, the National Institute of Mental Health (NIMH) Research Domain Criteria (RDoC) identified approach motivation as a fundamental dimension of behavior that cuts across diagnostic boundaries (5). Within RDoC, the Positive Valence Systems domain outlines multiple components of motivation including reward responsiveness, reward expectancy, reward valuation, effort valuation/willingness to work, and action selection/preference-based decision making (6). Reward responsiveness and reward expectancy refer to the in-the-moment and anticipated pleasure processes that occur in response to rewarding stimuli and cues. These hedonic processes help form internal representations of pleasure that serve to inform the appraisal and assignment of value to prospective rewards. The valuation of reward is then weighed against an effort-cost computation to determine if the reward is worth the effort as well as one's willingness to work for that reward. This cost-benefit analysis serves to guide not only decision making in the context of multiple choices, but also the subsequent execution of an action plan toward a final goal. These components align closely with frameworks that have emerged for the conceptualization of motivation as it pertains to psychiatric illnesses including SZ and MDD. For instance, in describing the motivational processes that contribute to goal-directed behavior, Barch and Dowd (7) suggest that hedonics or “liking” (i.e., reward responsiveness), and reward prediction and “wanting” (i.e., reward expectancy) interact to inform both reward valuation and effort valuation, leading to the development and implementation of an action plan (i.e., action selection/preference-based decision making) in order to achieve a desired goal.

To date, most studies examining motivation in SZ and MDD have focused on isolated facets of the motivation framework, typically within a single illness population. Accordingly, findings have emerged from behavioral and neuroimaging studies to suggest that patients with SZ exhibit impairments in reward valuation, reward expectancy, effort valuation, and action selection/decision making, but intact reward responsiveness (7–9). In contrast, patients with MDD have been shown to demonstrate impaired reward responsiveness, effort valuation, and action selection/decision making (8, 10). Given the heterogeneous nature of these illnesses, however, it is perhaps not surprising that these findings have been inconsistent across studies [see (8) for a review], with discrepancies often attributed to differences in cognitive functioning (i.e., memory, recall), medication status (e.g., atypical vs. typical antipsychotics), and symptom severity (i.e., anhedonia, negative symptoms) across clinical samples (8, 11–17). With schizotypal and depressive traits in non-clinical populations positioned on continua that extend to their respective clinical populations (18–20), investigating the multi-faceted motivation system in a non-clinical sample may afford insights into some of the processes related to clinical amotivation in schizophrenia and depression, while minimizing these potential illness-related confounds (21).

In addition to the heterogeneity within these patient samples, the variable findings across studies may also be a reflection of the multi-faceted nature of motivation itself, whereby impairments in any one or more of its underlying components can lead to the presentation of motivation deficits. To this end, these studies may be limited by their reliance on variable-centered approaches to examining isolated facets of motivation in that they do not address the heterogeneity existing within diagnostic groups, and fail to account for the possibility of different subtypes of motivation deficits. With this in mind, comprehensively examining the multiple components of motivation and adopting a person-centered approach may be well-suited to capture these individual differences, and resolve the multi-component heterogeneity of motivation (22). To date, however, no single study has yet to concurrently examine the multiple facets that comprise the motivation system in a population of healthy young adults, with only two studies, to our knowledge, examining effort valuation in a non-clinical sample (23, 24), and the majority of the remaining literature focusing on anhedonia (i.e., impaired reward responsiveness) in separate subclinical schizotypal or depressive samples (23, 25–27). Further, the extent to which the multiple facets of the motivation system interact along a continuum of natural variations in levels of schizotypal and depressive symptoms remains unknown.

Thus, the present study aimed to comprehensively explore dimensions of the multi-faceted motivation and reward system in a healthy undergraduate sample using a person-centered approach. Specifically, we sought to identify individuals based on similarities in performance across motivation tasks, hypothesizing the emergence of underlying homogeneous subgroups with differential motivation profiles. We subsequently evaluated differences between these behavioral profiles on measures of subclinical schizotypal and depressive symptoms. In addition, we investigated the inter-relationships between motivation facets, as well as their demographic, clinical, and cognitive correlates in order to elucidate the processes involved in performance on these tasks.

## Materials and methods

### Participants

A total of 117 undergraduate students at the University of Toronto Scarborough were recruited for this study through an online experiment registry for students in undergraduate psychology courses, and voluntarily participated for course credit. Participants were between the ages of 17–32, fluent in English, and capable of providing informed consent. Individuals were excluded from participation if they reported: taking any psychotropic medications; a current or past diagnosis of a schizophrenia-spectrum illness; a history of substance abuse or dependence within the past 6 months; or a history of neurological disease. This study was carried out in accordance with the recommendations of the Tri-Council Policy Statement, University of Toronto Research Ethics Board. All subjects gave written informed consent in accordance with the Declaration of Helsinki. The protocol was approved by the University of Toronto Research Ethics Board. Participation in the study involved a single visit to a psychology laboratory at the university where an experimenter provided instructions for, and administered a series of computerized self-report and behavioral measures.

### Measures

#### Self-report measures

All participants were administered the Personality Assessment Inventory (PAI), a 344-item diagnostic assessment to screen for psychopathology (28), the self-report version of the Apathy Evaluation Scale [AES-S; (29)] as a measure of clinical amotivation, and the Temporal Experience of Pleasure Scale [TEPS; (30)] to evaluate consummatory (TEPS-Con) and anticipatory (TEPS-Ant) pleasure. The Likert-scale version of the Schizotypal Personality Questionnaire [SPQ; (31, 32)] was administered to assess for schizotypal traits, from which positive (SPQ-Pos), negative (SPQ-Neg), and disorganized (SPQ-Dis) schizotypy subscores were also calculated. In addition, depressive symptoms were assessed using the Centre for Epidemiologic Studies–Depression Scale [CES-D; (33)]. Lastly, global cognitive functioning was assessed using the Brief Neurocognitive Assessment, consisting of tests of working memory and processing speed (34).

#### Objective computerized measures

A series of objective computerized tasks, informed by the RDoC Positive Valence System matrix (35), were subsequently administered to measure each facet of the motivation system. Participants were informed that all monetary rewards were hypothetical, but were instructed to play as though they would actually receive the money. For each task, outcome variables were converted into a single standardized z-score to represent the separate facets of motivation. Task administration was also randomized to minimize order effects. Only brief explanations of each task are presented below given that detailed descriptions, including specific instructions, have been previously published by their respective authors.

#### Reward responsiveness

**Figure 1:**
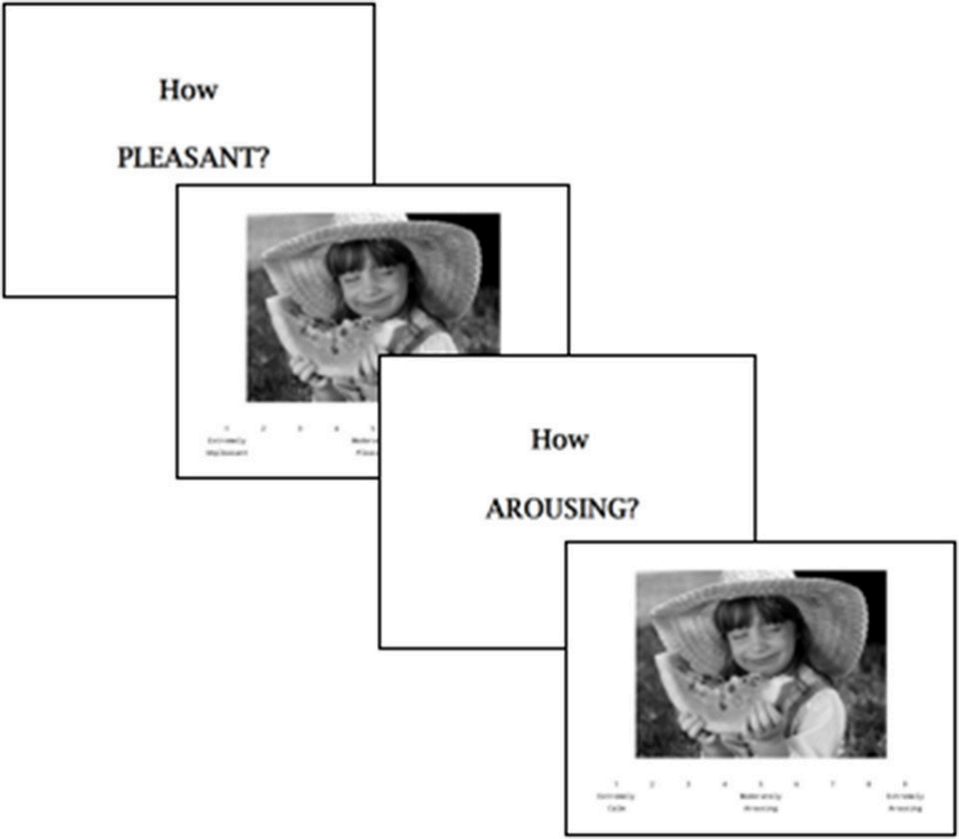
Schematic representation of a single trial in the International Affective Picture Rating System (IAPS) task (36). In this example, pleasantness and arousal ratings must be made for a positive-valence image.

The International Affective Picture System (IAPS), which has been extensively used in a number of neuropsychiatric illnesses including SZ and MDD, and in healthy adult samples (36, 37), was administered to assess for reward responsiveness or “liking” (36). In the current study, participants were presented with 42 positive, neutral and negative valence images^1^ (14 each), and instructed to rate in-the-moment pleasantness (IAPS-Pleasant) and arousal (IAPS-Arousal) on a 9-point Likert scale (Figure 1). Given our interest in approach motivation and reward-driven processes, we focused our analyses on positive-valence images only, with higher pleasantness and arousal ratings reflecting greater capacity to experience pleasure in response to positive stimuli. A composite score of the standardized IAPS-Pleasant and IAPS-Arousal variables was computed as a measure of reward responsiveness (IAPS-Composite).

#### Reward expectancy

**Figure 2:**
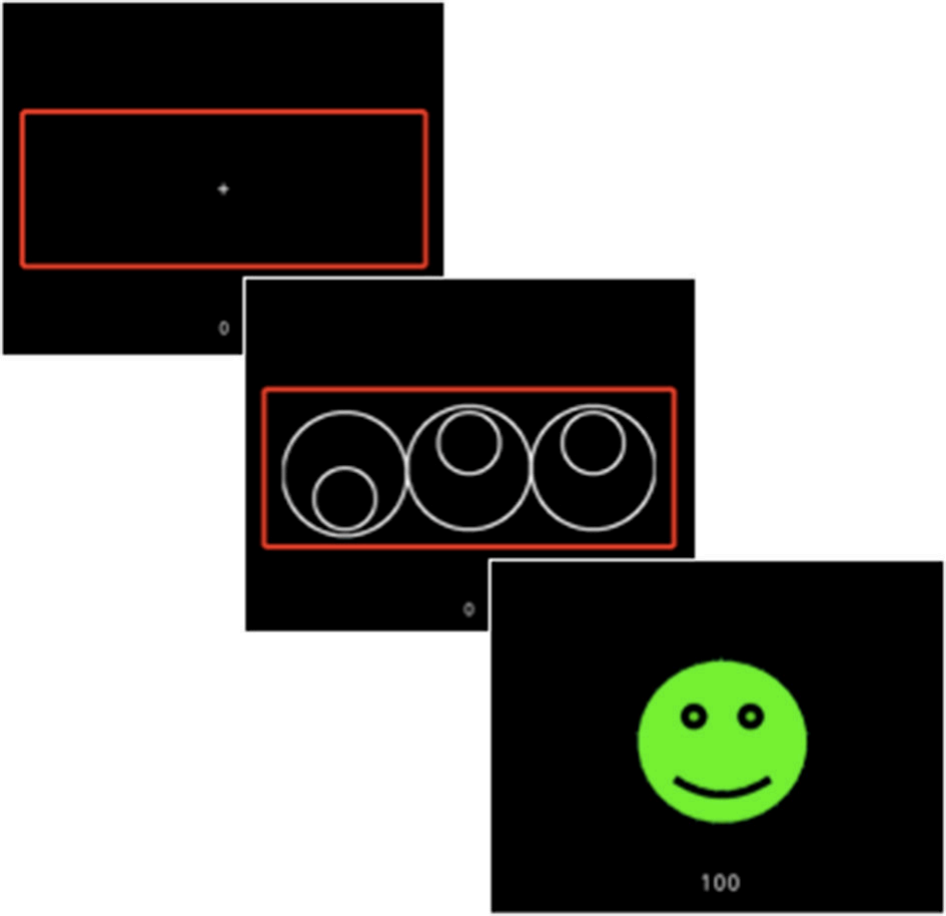
Schematic depicting the trial sequence in the Cued Reinforcement Reaction Time (CRRT) task (38). In this example, 100 points are awarded for a fast response on a high probability reinforcement trial.

The Cued Reinforcement Reaction Time (CRRT) task was used to assess for reward expectancy and “wanting” (38). The CRRT has been administered in SZ and healthy control (HC) samples (38, 39). Briefly, the CRRT requires rapid “odd-one-out” judgments on three circles across 96 trials. In each trial, the presentation of the circles is preceded by a cue with different colored (i.e., red, blue, or yellow) frames (Figure 2). Unbeknownst to the participant, each colored frame represents a differentially reinforced cue, such that points are awarded for correct responses on 90% (red frame), 50% (blue frame), and 10% (yellow frame) of the trials. However, participants are informed that the likelihood of receiving points is predetermined according to the color of the frame, and that they will receive 100 points for fast and correct responses, 1 point for slow and correct responses, and 0 points for incorrect responses. Accordingly, individuals should respond fastest when they anticipate a reward, particularly on the highest reinforcement probability (i.e., 90%) trials. Two practice blocks are administered before the task with the first to ensure task comprehension, and the second for calibrating individual reaction times. Specifically, the cut-off for a fast response was individually determined for each participant by subtracting one standard deviation from their median reaction time (RT) in the second practice block. The variable of interest is anticipated reinforcement-related speeding (CRRT-RRS) which represents the degree to which participants modulate their behavior (i.e., reaction time) in response to reward cues. CRRT-RSS was calculated by subtracting the median RT for low probability trials from the median RT for high probability trials, with smaller values are indicative of higher levels of RRS, or a greater degree of wanting.

#### Reward valuation

**Figure 3:**
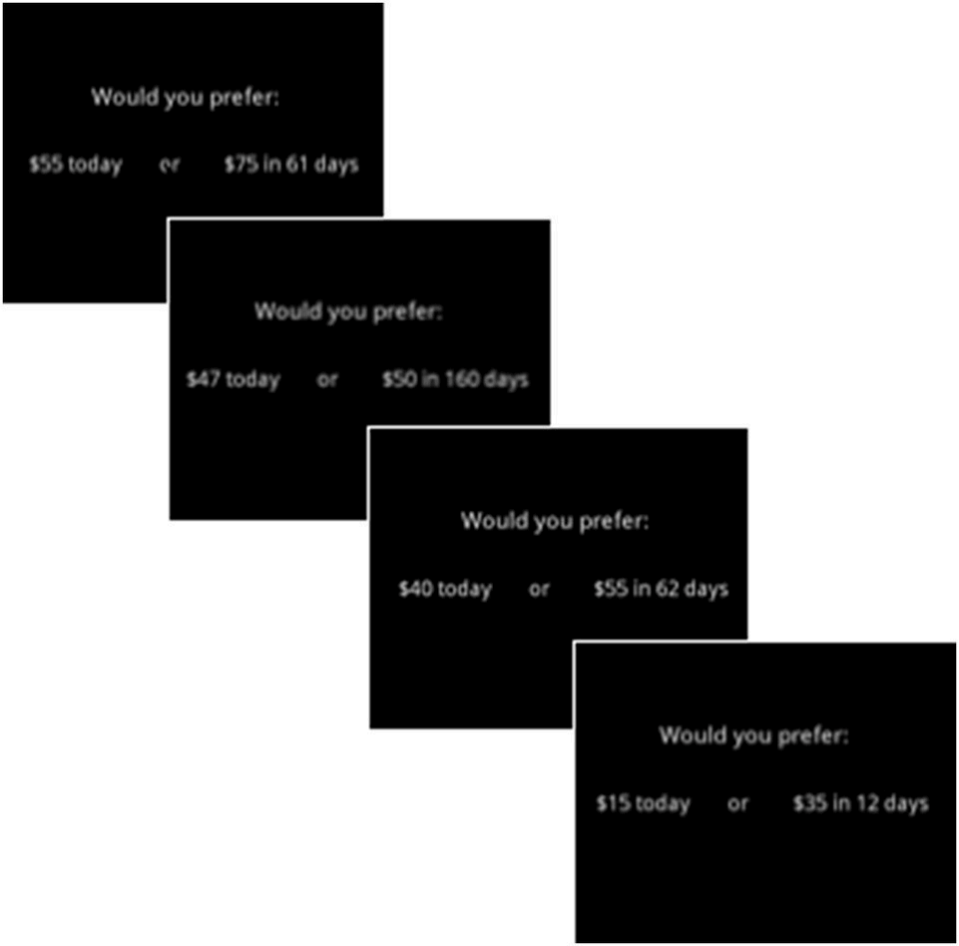
Examples of items utilized in the Kirby Delay Discounting questionnaire [Kirby DD; (40)].

Reward valuation was assessed using the Kirby Delay Discounting (Kirby DD) task which measures participants' differential valuation of monetary rewards over time (40). The Kirby task has been used in a number of neuropsychiatric disorders, including SZ and MDD, as well as in healthy undergraduate samples (41, 42). In this task, participants are instructed to choose between a small immediate reward and a larger delayed reward (over varying number of days) across 27 trials (Figure 3). The outcome of interest is the rate at which participants discount the value of future rewards, calculated as the average natural logarithm (*ln*) of the discounting rate (Kirby–Avg). Smaller values are indicative of lower discounting rates, or greater reward valuation.

#### Effort valuation

**Figure 4:**
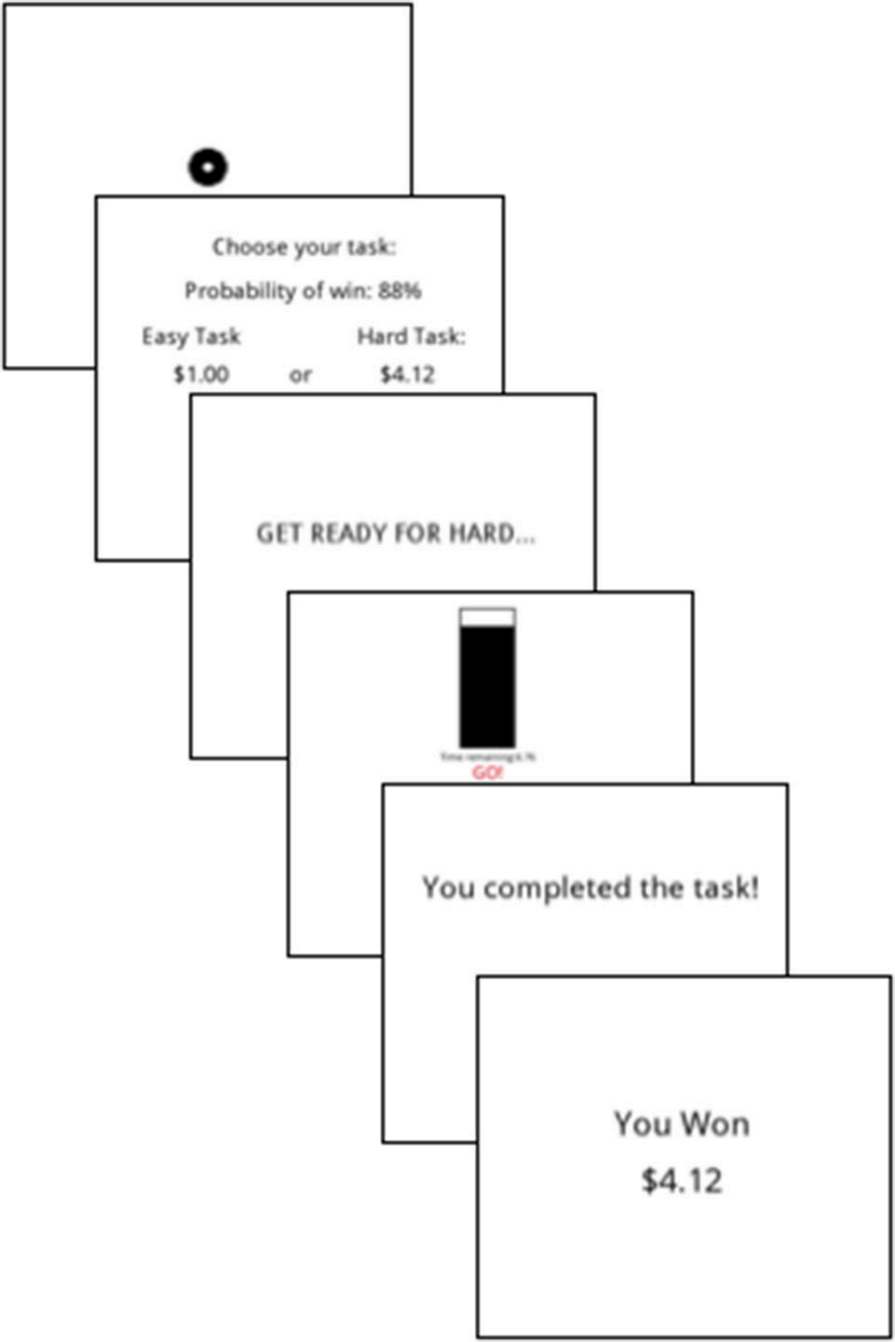
Schematic depicting the trial sequence in the Effort Expenditure for Rewards Task [EEfRT; (24)]. In this example, the hard task was selected and successfully completed on a high reward magnitude and high probability trial.

Effort valuation was assessed using the Effort Expenditure for Rewards Task (EEfRT), which measures participants' willingness to expend effort for monetary gains (24). The EEfRT is one of the most commonly used measures of effort-based decision making, and has been used in SZ, MDD, and undergraduate samples (23, 24, 43, 44). Participants are presented with the opportunity to receive a reward by choosing between two effortful button-pressing tasks that differ in level of difficulty. Specifically, the easy task requires 30 button presses (in 7 s) with the index finger of the dominant hand, whereas the hard task requires 100 button presses (in 21 s) with the pinky finger of the non-dominant hand. For each trial, participants are informed of the magnitude of the reward for the easy vs. hard task (ranging from $1.00 to $4.85), as well as the probability of receiving that reward (i.e., 12, 50, or 88%) if they successfully complete the task (Figure 4). On reinforced trials, the reward for successfully completing the easy task was always $1.00, whereas the reward for a successful completion of the hard task ranged from $1.24 to $4.21. Thus, for each trial, participants must compute a cost-benefit analysis based on task difficulty, reward magnitude and reward probability, which ultimately serves to guide their decision to expend effort. Given the impairments typically observed in clinical populations at the high probability condition, we specifically examined the proportion of hard tasks chosen at the 88% reward level (EEfRT-High), thus evaluating willingness to work when the likelihood of reward is most certain and least ambiguous.

#### Action selection/decision making

**Figure 5:**
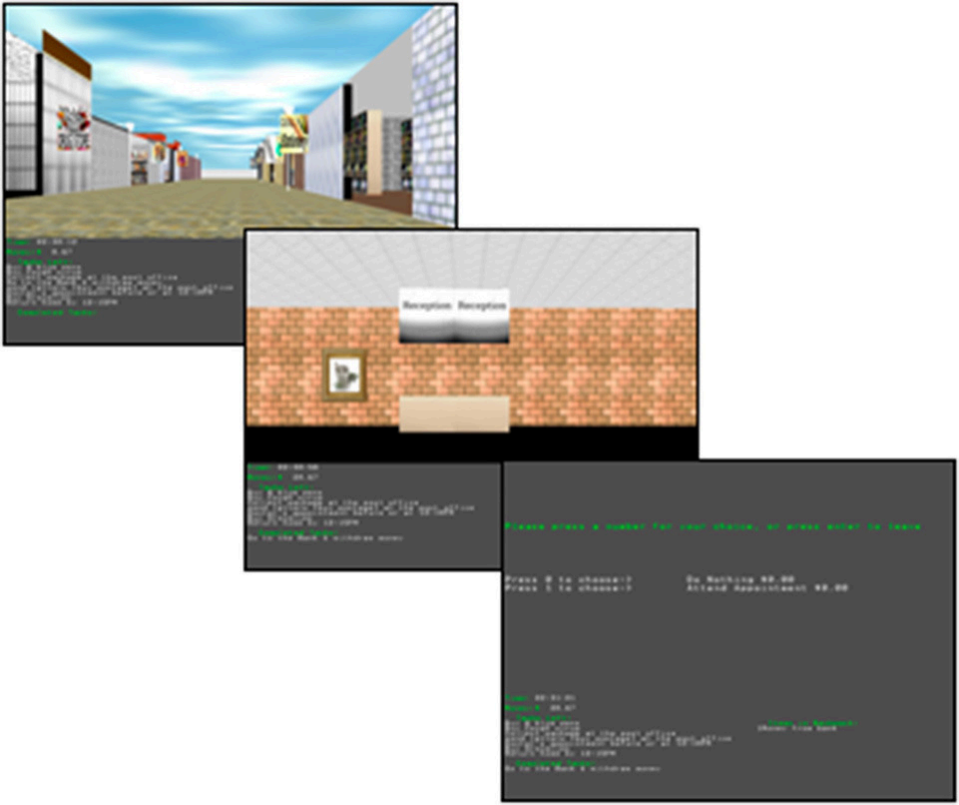
Screenshots of the virtual city in the Multitasking in the City Test [MCT; (45)].

Lastly, action selection/decision making was assessed using the Multitasking in the City Test [MCT; (45)], a virtual reality errand-running task which has been used in SZ (46) and non-clinical samples (45). In this task, participants are provided a map of the virtual city, as well as a list of eight pre-specified errands (e.g., buy groceries, attend a doctor's appointment, etc.) that must be completed within a period of 15 min. Participants navigate through the city using a joystick and are required to visit different stores in order to complete the required errands (Figure 5). Throughout the task, the list of completed and uncompleted errands is displayed on-screen to minimize the cognitive load. Participants are also provided feedback after each attempt at completing an errand, and must utilize this information to guide efficient decision making and goal-directed behavior (47). The MCT outcome variables are the total distance traveled (MCT-Distance; in virtual environment units—VEUs) and performance score (MCT-Performance), calculated as the total number of tasks completed minus total omission and commission errors (i.e., tasks failed or repeated, respectively). Shorter distances and higher performance scores indicate better decision making and planning. With MCT-Performance reflecting the decision making process, and MCT-Distance as an index of action selection and implementation, an MCT composite score was computed to represent the unified construct of action selection/decision making.

All self-report scales and objective motivation tasks were programmed and administered on Open Sesame v. 2.9.4 (48), except for the MCT which was run on a stand-alone software for Microsoft Windows. All computer tasks were presented on a 30′′ LCD display, with the participant providing input for each task either with the keyboard (for the IAPS, EEfRT, etc.) or with a standard video game controller (MCT).

### Analyses

#### Validity

Participants with clinically significant scores on any of the PAI clinical scales, as per Morey's guidelines (28) were excluded from all subsequent analyses (*n* = 5). Further, due to technological problems, an additional 16 participants with incomplete data in one or more of the motivation task were excluded, bringing the final sample to 96 participants. Sample demographics and symptom characteristics are shown in Table 1.

**Table 1:** Sample demographics and symptom characterization.

	**Mean (SD) *n* = 96**	**Range**
Sex (M:F)	38:58	–
Age	19.8 (2.4)	17.0–32.0
Education (yrs)	13.5 (1.3)	11.0–17.0
AES-S	30.7 (5.7)	21.0–49.0
CES-D	15.5 (10.1)	0.0–41.0
SPQ Total	115.4 (35.1)	14.0–207.0
SPQ Positive	45.9 (17.4)	0.0–79.0
SPQ Negative	43.1 (17.0)	1.0–81.0
SPQ Disorganization	26.4 (11.0)	0.0–51.0
TEPS-Con	4.6 (0.7)	2.8–6.0
TEPS-Ant	4.6 (0.7)	2.3–5.9
BNA Global	−0.13 (0.8)	−2.1–2.1
DCT	10.5 (2.2)	6.0–18.0

*AES-S, Apathy Evaluation Scale–Self-report version; CES-D, Centre for Epidemiologic Studies-Depression Scale; SPQ, Schizotypal Personality Questionnaire; TEPS-Con, Temporal Experience of Pleasure-Consummatory subscale; TEPS-Ant, Temporal Experience of Pleasure-Anticipatory subscale; BNA, Brief Neurocognitive Assessment; DCT, Dot Counting Test*.

In addition, the Dot Counting Test (DCT) was administered to assess for performance validity, such that participants with missing DCT data (*n* = 9), or a score greater than or equal to 14 (*n* = 10) were deemed invalid (49, 50). In an effort to conserve statistical power, analyses were first conducted with all 96 participants (results shown). The validity of these results was then confirmed by repeating the analyses (*n* = 77) after excluding participants who exerted non-credible findings.

#### Statistical analyses

Cronbach's alpha was calculated for each self-report measure to ensure consistent responding across the entire sample. Cluster analysis, using the k-means algorithm, as per MacQueen's methodology (51) was performed on reward responsiveness (i.e., IAPS-Composite), reward expectancy (i.e., CRRT-RRS), reward valuation (i.e., Kirby-Avg), effort valuation (i.e., EEfRT-High), and action selection/decision making (i.e., MCT-Composite) in order to identify unique subgroups of individuals with similar motivation task performance. Cluster solution was determined using the “NbClust” package, which utilizes an exhaustive array of indices (e.g., Silhouette, Calinski, and Harabasz index, etc.) to determine the optimal number of clusters (52). The consistency of the cluster solution was confirmed by establishing consensus with a second clustering method (i.e., hierarchical agglomerative clustering). Independent samples *t-*tests were subsequently conducted to assess for differences in clinical measures and motivation performance between clusters. Lastly, inter-relationships between motivation facets, and bivariate relations between clinical measures and facets of motivation were examined using Spearman correlations due to non-normal variable distributions.

All analyses were conducted using the Statistical Package of Social Science (SPSS) version 24, with the exception of the cluster analysis which was conducted using the “fpc” package version 2.1-10 (53) in R version 3.3.3 (54).

## Results

### Consistency of self-report clinical measures

Cronbach's alpha for the AES (α = 0.77), CES-D (α = 0.89), SPQ (SPQ-Pos: α = 0.90; SPQ-Neg; α = 0.92; SPQ-Dis: α = 0.90), and TEPS-Ant: α = 0.73 revealed good reliability across scales, suggestive of consistent responding amongst participants. The reliability of TEPS-Con was lower with an alpha coefficient of 0.63, though still similar to other studies utilizing this scale in a non-clinical sample (30, 55–57).

### Cluster analysis

**Figure 6:**
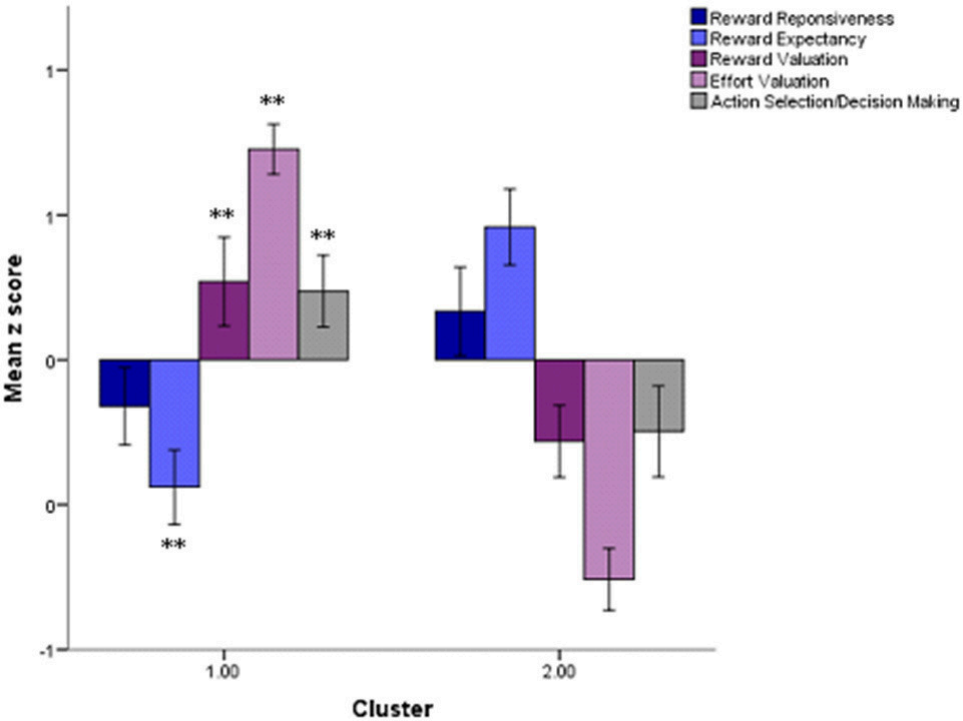
Motivation performance profiles across clusters. Significant differences are denoted by ^**^p < 0.01. Error bars represent standard error of the mean.

The k-means clustering algorithm identified a 2 cluster solution with 49 participants assigned to cluster 1 and 47 to cluster 2. The consistency of the cluster solution was verified using hierarchical clustering which revealed an 83% overlap between the two methods. Significant group differences reveal impaired reward expectancy in cluster 1, with cluster 2 characterized by impaired reward valuation, effort valuation, and action selection/decision making (Table 2). In contrast, the clusters did not significantly differ on performance in reward responsiveness. The motivation performance profiles of the clusters are shown in Figure 6. Additionally, as shown in Table 2, clusters did not significantly differ by age, sex, level of education, or consummatory or anticipatory pleasure. Clinically, there were no differences in severity of amotivation, overall schizotypal traits, or in depressive symptoms, though there was a non-significant trend for differences in negative schizotypy (*p* = 0.06). Importantly, excluding non-credible participants did not change these results.

**Table 2:** Demographic, clinical, and motivation performance characterization of clusters.

	**Cluster mean (SD)**				
	**1 (*n* = 49)**	**2 (*n* = 47)**	**t / χ^2^**	***p***	**Cluster diff**.
**DEMOGRAPHIC AND CLINICAL MEASURES**					
Age	19.6 (1.5)	20.1 (3.1)	−1.0	ns	–
Sex (M:F)	23:26	32:15	2.3	ns	–
Education	13.5 (1.4)	13.5 (1.3)	0.002	ns	–
AES-S	31.2 (5.5)	30.1 (5.8)	1.0	ns	–
CES-D	16.7 (10.0)	14.2 (9.8)	1.2	ns	–
SPQ-Total	118.9 (35.5)	111.7 (34.7)	1.0	ns	–
SPQ-Pos	46.0 (17.3)	45.8 (17.7)	0.05	ns	–
SPQ-Neg	46.3 (17.4)	39.8 (16.0)	1.9	.06	–
SPQ-Dis	26.6 (10.9)	26.1 (11.3)	0.2	ns	–
TEPS-Con	4.6 (0.7)	4.7 (0.7)	−0.5	ns	–
TEPS-Ant	4.5 (0.7)	4.7 (0.7)	−1.2	ns	–
BNA	−0.1 (0.9)	−0.2 (0.8)	0.4	ns	–
**MOTIVATION MEASURES**					
Reward responsiveness^a^	−0.2 (1.0)	0.2 (1.0)	−1.6	ns	–
Reward expectancy^b^	−0.4 (0.9)	0.5 (0.9)	−4.9	<0.001^bc^	1 > 2
Reward valuation^c^	0.3 (1.1)	−0.3 (0.8)	2.8	0.006^bc^	1 < 2
Effort valuation^d^	0.7 (0.6)	−0.8 (0.7)	10.9	<0.001^bc^	1 < 2
Action selection/decision making^e^	0.2 (0.9)	−0.2 (1.1)	2.4	0.017	1 < 2

bc *Denotes significance after Bonferroni correction for motivation test comparisons. [In addition to abbreviations defined in Table 1*,

a *was assessed using the International Affective Picture System (IAPS-Composite)*;

b *Using the Cued-Reinforcement Reaction Time Task (CRRT-RRS)*;

c *Using the Kirby Delay Discounting task (Kirby-Avg)*;

d Using the Effort Expenditure for Rewards Task (EEfRT-High); and

e *Using the Multitasking in the City Test (MCT-Composite)]*.

### Correlational analyses

Bivariate correlations between clinical measures and motivation task performance are shown in Table 3. Interestingly, AES, CES-D, and SPQ-Neg were only correlated with reward responsiveness as measured by the IAPS-Composite score. Reward responsiveness was also positively correlated with both TEPS-Con and TEPS-Ant.

**Table 3:** Bivariate correlations between clinical measures and motivation tasks.

	**Reward reponsiveness^a^**	**Reward expectancy^b^**	**Reward valuation^c^**	**Effort valuation^d^**	**Action selection/Decision making^e^**
Age	0.14	0.17^f^	0.06	0.10	−0.01
AES	−0.30^**^	0.05	0.04	0.12	−0.07
CES-D	−0.23^*^	−0.03	−0.03	0.08	0.15
SPQ Total	−**0**.18^f^	−0.12	−0.03	0.17	−0.09
SPQ Pos	0.09	−0.04	−0.15	0.15	−0.02
SPQ Neg	−0.33^**^	−0.08	0.10	0.16	−0.01
SPQ Dis	−0.14	−0.10	−0.03	0.07	0.02
TEPS-Con	0.25^*^	0.03	0.06	0.09	0.11
TEPS-Ant	0.45^**^	0.02	0.09	−0.04	−0.23^*^
BNA	0.02	−0.08	0.11	−0.10	0.09

Significant correlations (in bold font) are denoted by

* p < 0.05, or

** *p < 0.01*;

f *denotes p < 0.1. See Table 1 and Table 2 for abbreviations*.

### Inter-task correlations

Correlations were also conducted to examine the interrelationships between motivation variables. As shown in Table 4, significant positive inter-task correlations were found between reward responsiveness and reward expectancy, and between reward valuation and effort valuation.

**Table 4:** Inter-task correlations.

	**2**	**3**	**4**	**5**
1. Reward responsiveness^a^	0.22^*^	0.02	−0.03	−0.14
2. Reward expectancy^b^	–	0.03	−0.15	0.10
3. Reward valuation^c^		–	0.21^*^	−0.10
4. Effort valuation^d^			–	0.14
5. Action selection/Decision making^e^				–

Significant correlations (in bold font) are denoted by

* *p < 0.05*.

*See Table 2 for abbreviations*.

## Discussion

Informed by the RDoC Positive Valence Systems domain (6), the present study aimed to comprehensively explore the multiple facets of the motivation and reward system in a non-clinical sample using a battery of objective computerized tasks. Drawing from dimensional approaches to understanding psychopathology (5), we specifically utilized a person-centered approach to identify subgroups of individuals based on motivation task performance. The results of our cluster analyses revealed two groups of individuals with distinct behavioral profiles, such that the first cluster demonstrated impairments in reward expectancy, whereas cluster 2 was characterized by impaired reward valuation, effort valuation, and action selection/decision making. Interestingly, however, reward responsiveness was comparable between clusters, perhaps a reflection of the relatively low levels of amotivation endorsed by these individuals. In fact, a study by our group examined anhedonia in SZ and HC participants and found that it was only the individuals with high levels of amotivation (i.e., AES scores > 40) who demonstrated impaired reward responsiveness (12). Thus, the absence of a cluster difference in reward responsiveness may in part be a result of the restricted severity of amotivation in the present sample, which may not translate into impairments in this specific facet of motivation. Further, clusters did not significantly differ in age, sex, cognitive functioning, depressive symptoms, or overall schizotypal traits. There was, however, a non-significant trend for differences in negative schizotypy between clusters, such that individuals in cluster 1 endorsed higher levels of negative schizotypal traits compared to those in cluster 2. Given that cluster 1 was characterized by impaired reward expectancy, this elevation in negative schizotypy seems to fit well with other studies similarly revealing reduced anticipatory pleasure in individuals with negative schizotypal symptoms (27, 58–60). Nonetheless, the absence of significant differences between clusters is particularly important as it suggests that individuals are not simply being clustered by symptom severity or cognitive functioning, but rather by similar performance on discrete facets of motivation. Interestingly, clusters also presented with comparable levels of clinical amotivation, further highlighting the multi-faceted nature and varying behavioral underpinnings of motivational impairments.

Correlational analyses revealed that reward responsiveness, was related to amotivation, negative schizotypal traits and depressive symptoms, such that higher scores on the IAPS was associated with lower symptomatology. These findings are in line with other studies that have demonstrated a relationship between overall negative symptoms and pleasure in non-clinical samples (25, 30, 61). Interestingly, the MCT composite score was also positively associated with anticipatory pleasure, perhaps indicative of the hedonic processes involved in preference-based decision making and goal-driven action selection. The overall lack of significant correlations between symptom measures and facets of motivation, however, may be a reflection of the lower symptom severity in this sample, with more robust and widespread relationships emerging in clinical populations such as SZ and MDD that experience more severe impairments.

Correlational analyses were also conducted to explore the inter-relationships between the objective motivation measures. Our results revealed a significant relationship between reward responsiveness (i.e., “liking”) and reward expectancy (i.e., “wanting”). These findings are in line with the literature on anhedonia in SZ and MDD which distinguishes between the separate, but inter-related consummatory and anticipatory components of hedonic experience (30, 62). Further, the significant correlation between reward valuation and effort valuation fits well with the current conceptualizations of the motivation framework, with reward valuation and effort valuation subsumed into one construct representing cost-benefit decision making (7). Thus, our findings lend further support to the inter-related reward and effort valuation processes that serve to inform cost-benefit analyses in the context of rewards and goals. Overall, however, the small and limited inter-correlations across these facets suggest that they are indeed distinct and are likely tapping into different domains of the motivation system.

There are limitations to this study that warrant mention. First, our study sample consisted entirely of relatively young university students, which limits the generalizability of these results to other populations. Further, although participants were excluded if they reported a history of psychiatric illness, a formal diagnostic interview was not conducted to confirm the absence of any current or past disorders. However, we did utilize the PAI, a valid self-report measure of psychopathology, and excluded participants with clinically significant scores. The subjective nature of symptom measures serves as another limitation of this study, though we did include objective measures of motivation to augment these clinical rating scales, and administered the DCT in order to minimize non-credible responding secondary to poor effort. In addition, our computerized measures of motivation utilized different types of intangible rewards including pleasant imagery, points, and hypothetical monetary reward; however, a number of studies have failed to find differences between hypothetical (i.e., abstract) and real (i.e., tangible) rewards both in terms of behavioral performance and resultant brain activity during these tasks (63–65). It is also important to note that cluster analyses, though widely used, are exploratory and no standard method exists for determining the optimal number of clusters. While subjective judgment is often required, we took additional steps to validate our results by establishing consensus amongst an exhaustive list of indices, and with a different clustering method. Moreover, while there are no statistical guidelines for calculating minimum sample size requirements to conduct cluster analysis, our sample size exceeded Formann and Kohlmann's (66) recommendation of a minimum sample size of 2^k^ where k equals the number of clustering variables. That said, it is still possible that our sample size may have restricted the number of clusters identified, and it will therefore be important to replicate our findings in larger groups. Finally, the comprehensive evaluation of discrete facets that comprise the motivational system would benefit from inclusion of multiple reward-based tasks believed to tap into overlapping constructs, although to our knowledge the present investigation represents the most extensive to date.

The findings of the present study revealed two clusters of individuals with differential motivation performance profiles. Going forward, it will be important to investigate the underlying neural circuits distinguishing these profiles. Though speculative, the reward anticipation deficits in cluster 1 may be related to striatal dysfunction (8, 67), with the impairments seen in cluster 2 potentially associated with higher-order cortical regions of the brain (8, 13). Further, it remains to be seen whether this pattern of results will be similar in SZ and MDD populations with more severe symptoms and motivational impairments. Our results also provide further support for the current conceptualization of motivation as a multi-faceted framework comprised of distinct, but interrelated components. Taken together, our findings align with recent dimensional approaches to understanding core domains of psychopathology, and highlight the need for continued comprehensive evaluations of the motivation system across a continuum of healthy and clinical populations.

## Author contributions

GF and AA: Designed the study; SD: Led the statistical analyses and preparation of the first draft of the manuscript; AA, SS, and IS: Contributed to the data collection and statistical analyses. All other authors subsequently made significant contributions to the interpretation of the findings, and have approved the final manuscript.

### Conflict of interest statement

ZJD has received research support from Brainsway Inc. and Magventure Inc., has served on the advisory board for Sunovion, Hoffmann-La Roche Limited and Merck, and has received speaker support from Eli Lilly. GR has received consultant fees from Neurocrine Biosciences and Synchroneuron, as well as research support from Novartis. GF has served as an investigator on research sponsored by Medicure Inc., and Neurocrine Bioscience. He has also served on advisory boards for Hoffman-La Roche and Takeda, and received speaker's fees from Hoffman-La Roche, Lundbeck and Novartis. The other authors declare that the research was conducted in the absence of any commercial or financial relationships that could be construed as a potential conflict of interest.

## Footnotes

^1^IAPS stimuli used in the study included: 1052, 1120, 1300, 1390, 1450, 1560, 1602, 1620, 2270, 2304, 2320, 2360, 2370, 2480, 2490, 2495, 2590, 2722, 4598, 5779, 5891, 5920, 5950, 5971, 6260, 6560, 7325, 7640, 8030, 8080, 8160, 8180, 8185, 8370, 8400, 8490, 9001, 9210, 9220, 9250, 9280, 9331.
